# Decreased renal cortical perfusion, independent of changes in renal blood flow and sublingual microcirculatory impairment, is associated with the severity of acute kidney injury in patients with septic shock

**DOI:** 10.1186/s13054-022-04134-6

**Published:** 2022-09-01

**Authors:** James Watchorn, Dean Huang, Kate Bramham, Sam Hutchings

**Affiliations:** 1 School of Immunology and Microbial Sciences, King’s College London, London, UK; 2 Academic Department of Military Anaesthesia and Critical Care, Royal Centre for Defence Medicine, Birmingham, UK; 3 Department of Radiology, King’s College Hospital NHS Foundation Trust, London, UK; 4 Department of Women and Children’s Health, King’s College London, London, UK

**Keywords:** Acute kidney injury, Septic shock, Microcirculation, Contrast-enhanced ultrasound, Echocardiography, Renal blood flow, Sublingual video microscopy

## Abstract

**Background:**

Reduced renal perfusion has been implicated in the development of septic AKI. However, the relative contributions of macro- and microcirculatory blood flow and the extent to which impaired perfusion is an intrinsic renal phenomenon or part of a wider systemic shock state remains unclear.

**Methods:**

Single-centre prospective longitudinal observational study was carried out. Assessments were made at Day 0, 1, 2 and 4 after ICU admission of renal cortical perfusion in 50 patients with septic shock and ten healthy volunteers using contrast-enhanced ultrasound (CEUS). Contemporaneous measurements were made using transthoracic echocardiography of cardiac output. Renal artery blood flow was calculated using velocity time integral and vessel diameter. Assessment of the sublingual microcirculation was made using handheld video microscopy. Patients were classified based on the degree of AKI: severe = KDIGO 3 v non-severe = KDIGO 0–2.

**Results:**

At study enrolment, patients with severe AKI (37/50) had prolonged CEUS mean transit time (mTT) (10.2 vs. 5.5 s, *p* < 0.05), and reduced wash-in rate (WiR) (409 vs. 1203 au, *p* < 0.05) and perfusion index (PI) (485 vs. 1758 au, *p* < 0.05); differences persisted throughout the entire study. Conversely, there were no differences in either cardiac index, renal blood flow or renal resistive index. Sublingual microcirculatory variables were not significantly different between groups at study enrolment or at any subsequent time point. Although lactate was higher in the severe AKI group at study enrolment, these differences did not persist, and there were no differences in either ScvO2 or ScvCO2-SaCO2 between groups. Patients with severe AKI received higher doses of noradrenaline (0.34 vs. 0.21mcg/kg/min, *p* < 0.05). Linear regression analysis showed no correlation between mTT and cardiac index (R-0.18) or microcirculatory flow index (R-0.16).

**Conclusion:**

Renal cortical hypoperfusion is a persistent feature in critically ill septic patients who develop AKI and does not appear to be caused by reductions in macrovascular renal blood flow or cardiac output. Cortical hypoperfusion appears not be associated with changes in the sublingual microcirculation, raising the possibility of a specific renal pathogenesis that may be amenable to therapeutic intervention.

*Trial Registration* Clinical Trials.gov NCT03713307, 19 Oct 2018.

## Background

Septic shock is one of the commonest causes of acute kidney injury (AKI) in critically ill patients and is associated with a mortality of approximately 50% [1, 2]. Septic AKI presents a unique pathophysiology; once considered a consequence of systemic circulatory insufficiency leading to acute tubular ischaemia, it is now widely accepted as being the result of multiple factors including inflammation, alterations in renal perfusion and changes in cellular bioenergetics [3–5]. However, the pathogenesis in humans remains elusive and difficult to study in critical illness.

Contrast-enhanced ultrasound (CEUS) is an emerging imaging technique in the field of critical illness using highly echogenic but inert microbubbles to delineate areas of microvessel perfusion within organs. When given by infusion, high-frequency ultrasound pulses destroy the microbubbles allowing the creation of time intensity curves during subsequent reperfusion. Both experimental data [6] and clinical studies [7–9] using CEUS have demonstrated that renal cortical perfusion is impaired in septic AKI. Ultrasound contrast agents (UCAs) are widely used, licensed pharmaceutical agents with rarely reported adverse events. Serious adverse events occur at a rate of 0.03% and anaphylactoid reactions occur approximately at a rate of 1 in every 11,000–25,000 patient exposures [10].

The aim of the current study is to examine renal cortical perfusion using CEUS in a larger cohort of patients with septic shock, alongside detailed measurement of cardiac output, renal blood flow and flow in the sublingual microcirculation in order to further develop our understanding of the pathogenesis of this important condition.

## Methods

### Study design

A single-centre prospective longitudinal observational study is carried out at a tertiary critical care centre in the United Kingdom. Serial assessments were made of the renal and systemic macro- and microcirculatory state using CEUS, transthoracic echocardiography and sublingual video microscopy at 4 time points: Day zero (D0) within 24 h of ICU admission, D0 + 24 h (D1), D0 + 48 h (D2) and D0 + 96 h (D4). The study protocol was published prior to data analysis [11].

Ethical approval was granted by Yorkshire and the Humber, Leeds West Research Ethics Committee (18/YH/0371).

### Diagnosis of AKI

AKI was staged using KDIGO criteria [12]. Common to AKI research, the baseline creatinine value may not be known and therefore to define AKI biochemically is not always possible. For those who had AKI defined using creatinine, we used the lowest value of any from within the 12 months prior to admission as a baseline value. If no preadmission creatinine was available, the lowest value the creatinine returned to after resolution of acute illness was used. Failing all the above and if AKI could not be staged by other criteria, the admission creatinine was used as the baseline value.

### Inclusion and exclusion criteria

Only adult patients (> 18 years) were included. Patients were also required to have met all of the following criteria: To be within 24 h of admission to the ICU, have evidence of suspected or confirmed infection a Serial Organ Failure Assessment (SOFA) score increase of 2 or more a vasopressor requirement to maintain a mean arterial blood pressure of 65 mmHg and an arterial blood lactate of > 2 mmol/L after initial fluid resuscitation.

Patients were excluded from the study if they had stage four chronic kidney disease or worse, a renal transplant, contraindication to SonoVue™ (Bracco SpA, Milan, Italy) contrast or if the intent of treatment was primarily palliative. Pulmonary hypertension, a relative contraindication to SonoVue, was measured with transthoracic echo prior to administration of contrast and withheld if the pulmonary artery systolic pressure was > 90 mmHg.

### Sample size

The sample size was derived from a power calculation, although the published CEUS data from critically ill patients was limited*.* Based on an estimated difference in perfusion index of 1000 au between groups (AKI present and AKI absent) and on a standard deviation (SD) of 1000 au and assuming a power of 90% and alpha of 0.05, 22 patients in each group (*n* = 44) were estimated to detect a difference in perfusion index, assuming approximately 50% of patients with septic shock develop AKI. In order to address potential for missing data, unequal groupings or refusal of retrospective consent, the population size was set at 50.

### Contrast-enhanced ultrasound

Renal ultrasound was performed using an Affiniti ultrasound system (Philips Medical Systems International B.V., Best, The Netherlands). After conventional grayscale US imaging using the Philips C5-1 transducer at the patient’s flank, any patient with ultrasonographic evidence of CKD was excluded and the most accessible kidney chosen to perform the study provided both kidneys appeared normal (defined as an absence of hydronephrosis, atrophy, cortical thinning or other abnormality identified by the investigator). Baseline greyscale and colour Doppler sonographic images were acquired and the renal resistive index measured in an intralobular artery as described elsewhere [13]. For CEUS imaging a low mechanical index (MI) was utilised (MI < 0.1) in dual image display format. This format displays two views in parallel; the contrast view is constructed by selectively filtering the signal, identifying microbubbles, while excluding background signal, as a result the initial image is blank prior to the infusion of contrast. The simultaneously displayed standard greyscale image allows the kidney to be identified prior to the infusion. Low MI ultrasound is necessary to prevent microbubble destruction as the contrast is inherently unstable when insonated. Once an acceptable and stable view had been established, an infusion of 4.8 mL of SonoVue contrast agent was administered by a dedicated infusion pump at a rate of 1 mL/min until the total volume had been infused. This method is identical to that described by previous authors [7, 14].

Images of the entire examination were digitally recorded. When delivered centrally, contrast typically arrives in the kidney at approximately 45 s, after which the kidney continues to enhance until approximately 1 min to 1 min 30 s. By 2 min a steady state of contrast enhancement was reliably achieved and the kidney was not further enhancing. Five high-frequency pulses were subsequently delivered, one every 30 s. Microbubbles are destroyed immediately on pulsing, followed by reperfusion of bubble contrast and these destruction-replenishment cycles were used to model perfusion-based indices.

**Fig. 1:**
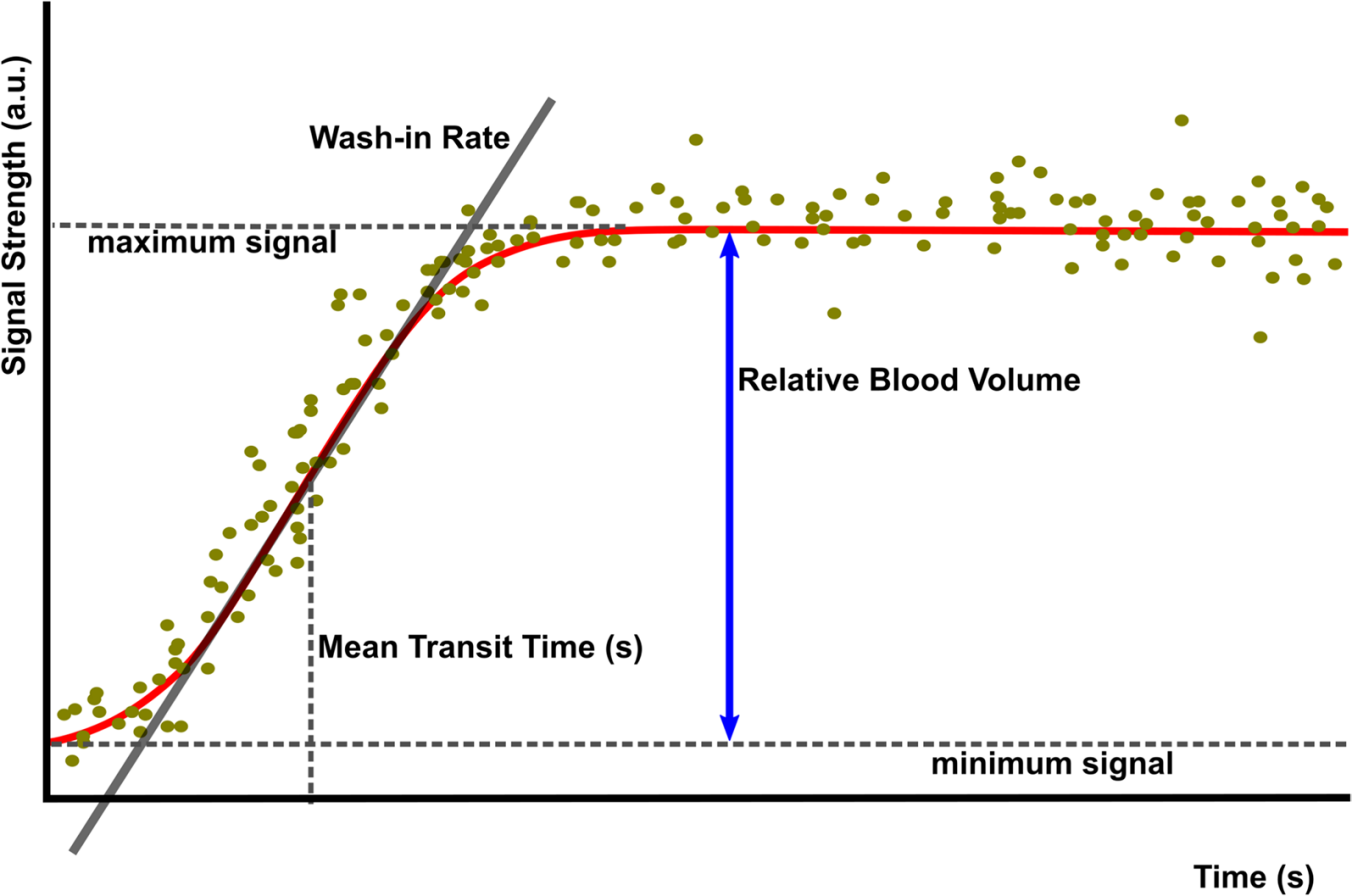
Schematic diagram of typical destruction-replenishment kinetics following the administration of ultrasound contrast

Post-processing was performed offline using VueBox™ (Bracco Diagnostic Imaging, Switzerland). Cortical regions lying in proximity to the probe with good views and reliably visible reperfusion were identified as regions of interest. Time intensity curves (TIC) were produced with several key components as shown in Fig. 1. Four variables were measured from the TICs, the mean transit time (mTT)—the time from nadir to half intensity; the wash-in rate (WiR)—the reperfusion gradient; relative blood volume (RBV)—the maximum minus the minimum signal intensity and the perfusion index (PI)—a composite measure calculated by RBV/mTT. Time-based variables are more representative of flow, while intensity-based variables are suggested to represent blood volume within a given region [15].

The plateau of peak intensity minus the background signal is termed the Relative Blood Volume (RBV) and is reflective of the total volume of the vascular space within the ROI. The maximal gradient of the wash-in slope is termed the wash-in rate (WiR) and the time taken to reach half-maximum signal intensity the mean transit time (mTT). Perfusion index (PI) is a composite variable produced by the division of RBV by mTT and aims to provide a single number representing tissue perfusion.

### Renal artery ultrasound

Following completion of the CEUS protocol the contrast infusion rate was reduced to 0.5 mL/min to provide more time and reduced intensity, which provided optimal images of the renal artery. The diameter of the renal artery was measured on a magnified contrast-enhanced view. Pulsed wave Doppler was then used to quantify time-averaged velocity (TAV) within the renal artery at the same location as the diameter measurement. TAV is the area under the Doppler time curve per pulsed waveform. Quantification of renal blood flow was calculated using the following equation:

$$RBF=\pi { \mathrm{renal} \mathrm{artery} \mathrm{radius}}^{2} \times \mathrm{TAV}\times \mathrm{HR} \times 2\times k$$

RBF: renal blood flow; πr [2] provides the area of the renal artery. TAV: time-averaged velocity within the renal artery; HR: heart rate; 2: assuming 50% RBF per kidney; k: correction factor (see discussion).

### Sublingual video microscopy

Videos of sublingual microcirculation were acquired using an Incident Dark Field (IDF) video-microscope (Cytocam, Braedius Medical, Huizen, The Netherlands). Acquisition and analysis of video sequences were carried out by a single operator in accordance with the most recent international consensus criteria [16].

### Transthoracic echocardiography

A comprehensive transthoracic echocardiogram study was performed in order to gather the minimum dataset required by the British Society of Echocardiography (BSE) [17]. A single, BSE accredited operator performed and analysed all the scans.

### Outcomes

The original intent, outlined in the published protocol was to analyse patients according to the presence or absence of AKI. However, the vast majority of the cohort exhibited at least some degree of AKI (KDIGO stage ≥ 1) ,and we therefore changed our primary outcome from that published in the protocol, prior to data analysis, based on the presence or absence of severe AKI (KDIGO 3 v KDIGO 0–2).

### Statistical analysis

Continuous data were examined for normality using the Shapiro Wilk test and parametric data reported as mean ± standard deviation (SD) and compared using the non-paired t test. Nonparametric data are reported as median and inter-quartile range (Q1–Q3) and compared using the Wilcoxon rank-sum test. Ordinal data was reported as number and percentage and compared using Chi-squared test. Longitudinal data were compared between individual days using analysis of variance and post hoc testing undertaken by Tukey’s Honest Significant Differences if normal or Kruskal–Wallis test if nonparametric. Longitudinal trends over time were assessed using regression analysis, either linear or local regression, stated within the results. Data were normalised by log or Cox-Box transformation where appropriate. All data were assessed using R version 4.0.3. All *p* values were two-sided and considered significant at < 0.05.

## Results

### Patient characteristics

Fifty-one patients were recruited, but one subsequently withheld consent for data analysis. Thirty-seven (74%) were classified as KDIGO stage 3, four (8%) stage 2, 2(4%) stage 1, and seven (14%) did not develop AKI. Provided the patients remained alive for the study duration, CEUS imaging was achieved in all and at all time points. There were no adverse events from contrast administration. In the severe AKI group, five patients had died by D1, six by D2 and ten by D4. In the non-severe group, no patients died. All conducted observations were included in the final analysis. Baseline characteristics of the study patients are shown in Table 1.

**Table 1:** Characteristics of patients at study enrolment based on the degree of acute kidney injury

	Severe AKI	Non-severe AKI	Control	Significance (*p*)
Age	63 (46–73)	67 (61–71)	34 (30–36)	0.21 patient groups; < 0.001 vs. controls
Female (%)	38	39	58	0.45
SOFA	15.3 ± 3.3	9.3 ± 1.9		< 0.001
Norepinephrine dose (mcg/kg/min)	0.34 (0.26–0.51)	0.21 (0.14–0.3)		0.009
Additional inotropes *n*(%)	18 (49)	1 (8)		0.02
Lactate (mmol/l)	3.9 (2.7–7.4)	2.7 (1.9–3.5)		0.03
Base Excess (mEq/l)	−6.3 ± 4.2	−4.0 ± 4.3		0.14
Haemoglobin (mg/dl)	93 (81–111)	108 (99–115)		0.07
C Reactive protein (mg/dl)	150 (99–279)	256 (160–293)		0.25
White cell count (× 10^9/L^)	11.0 (7.1–25.6)	13.2 (9.4–14.5)		0.92
PcvCO2-PaCO2 (kPa)	0.89 (0.54–1.16)	0.875 (0.748–1.11)		0.59
ScvO2 (%)	68 ± 14.4	70 ± 7.8		0.72
Mean Arterial Pressure (mmHg)	69 (-62–74)	70 (68–73)		0.84
Heart rate	100 ± 18	91 ± 15		0.11
CKD stage 2–3 (*n*)	5	0		0.40
Hypertension (*n*)	12	7		0.27
Diabetes (*n*)	9	4		0.89

*SOFA* Serial Organ Failure Assessment, *ScvO2* central venous oxygen saturation, *PcvCO2* central venous partial pressure carbon dioxide, *PaCO2* arterial partial pressure carbon dioxide

Thirty-three patients (89%) in the severe AKI group were diagnosed as such within the first 24 h of admission, the remaining four patients were diagnosed over the following four days of the study period. Thirty (81%) of the severe AKI group received renal replacement therapy within the first 24 h of admission and of those 30 patients, 26 could also have been classified as stage 3 AKI by either urinary or biochemical measurements taken within in the day of ICU admission.

The suspected site of infection was predominantly from gastrointestinal perforation or infarction (31%); other causes included lower respiratory tract infection (26%) and urological sepsis (8%). No site was identified in 13%. No causative organisms were grown in 35% of cases. In the remainder, gram negative bacteria predominated (33%), followed by gram positive bacteria (20%) and fungal infection (8%).

### Renal cortical perfusion

**Fig. 2:**
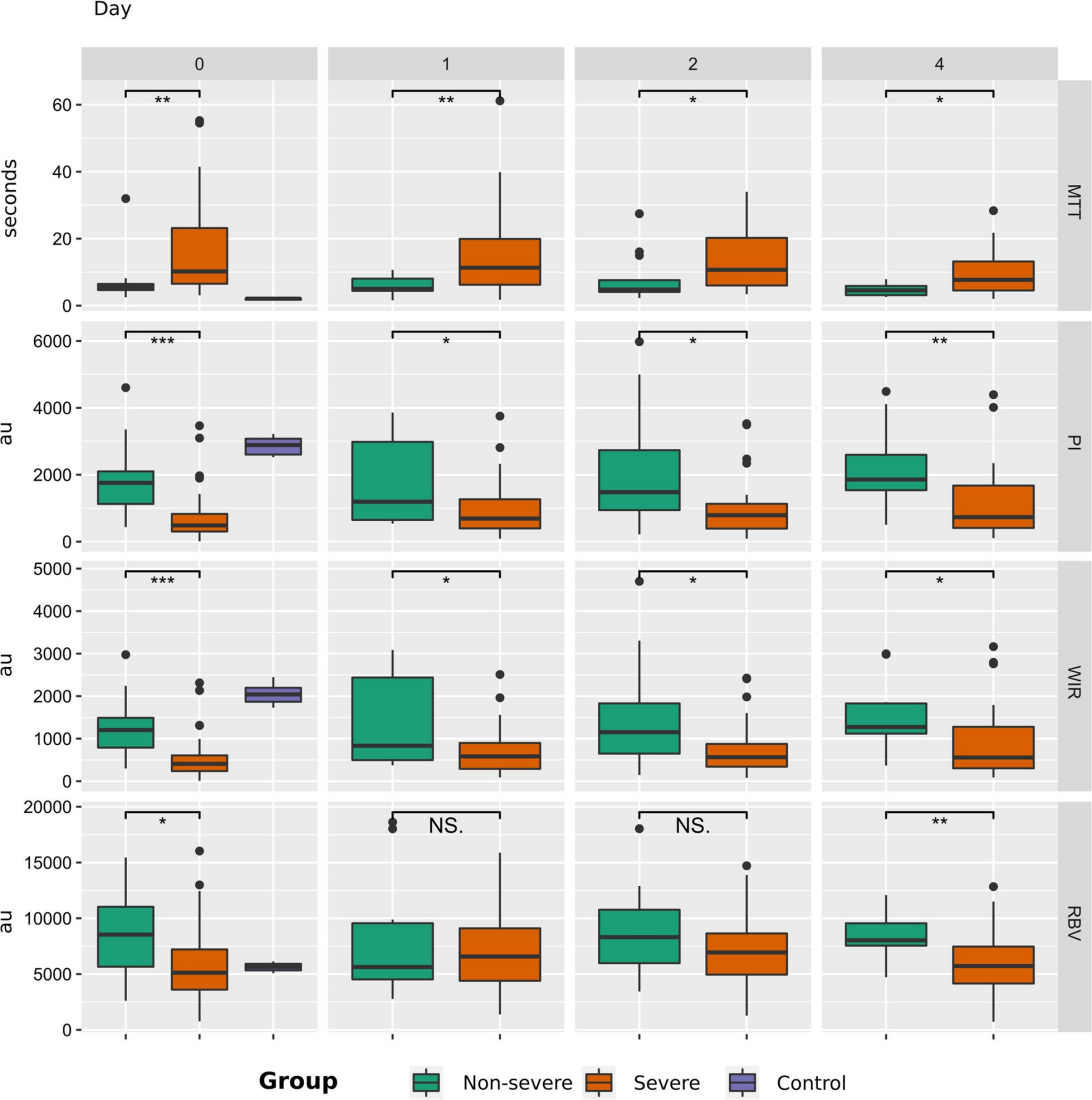
Renal cortical perfusion assessed by CEUS variables, presented over time and between severe AKI, non-severe AKI and control groups

Cortical CEUS parameters for healthy volunteers and patients with and without severe AKI are shown in Fig. 2 and Table 2. Patients with septic shock had significantly worse renal cortical perfusion than healthy volunteers, evident in all CEUS variables that measure blood flow against time (Mean Transit Time, Wash-in Rate and Perfusion Index). CEUS Renal Blood Volume was reduced in healthy volunteers compared to septic shock patients without severe AKI, but not in those with severe AKI.

**Table 2:** CEUS parameters for healthy controls and patients with severe AKI and non-severe AKI, between groups and within groups over time

Timepoint	D0	D1	D2	D4	Significance (*p*)
*mTT (s)*					
HC	2.09 (1.78–2.14)				
Severe	10.2 (6.54–23.2)	11.3 (6.24–19.9)	10.7 (6.02–20.2)	7.7 (4.54–13.2)	*0.11*
Non-severe	5.54 (4.65–6.47)	5.03 (4.43–8.06)	4.81 (4.1–7.61)	4.57 (3.14–5.88)	*0.45*
*significance*	< *0.05 (all groups)*	< *0.05*	< *0.05*	< *0.05*	
*WiR (a.u.)*					
HC	2040 (1868–2196)				
Severe	409 (238–606)	583 (291–899)	555 (342–876)	558 (304–1278)	*0.21*
Non-severe	1203 (790–1489)	833 (496–2438)	1152 (649–1830)	1274 (1119–1829)	*0.90*
*Significance*	< *0.05 (all groups)*	< *0.05*	< *0.05*	< *0.05*	
*PI (a.u.)*					
HC	2888 (2603–3077)				
Severe	485 (302–829)	690 (396–1266)	791 (390–1130)	732 (410–1676)	*0.36*
Non-severe	1758 (1126–2100)	1193 (649–2983)	1479 (942–2734)	1856 (1538–2595)	*0.89*
*Significance*	< *0.05 (all groups)*	< *0.05*	< *0.05*	< *0.05*	
*RBV (a.u.)*					
HC	5644 (5337–5917)				
Severe	5123 (3604–7209)	6572 (4397–9102)	6940 (4950–8642)	5722 (4155–7463)	*0.18*
Non-severe	8538 (5659–11,030)	5630 (4523–9558)	8308 (5978–10,770)	8025 (7549–9553)	*0.95*
*Significance*	< *0.05 (severe vs. non-severe)*	*0.97*	*0.43*	< *0.05*	

Italics are used to identify the significance values

*mTT* mean transit time, *WiR* wash-in rate, *PI* perfusion index, *RBV* relative blood volume, *D0* day 0, *D1* day 1, *D2* day 2, *D4* day 4

### Systemic and regional heamodynamic variables

Systemic and regional haemodynamic measurements for patients with and without severe AKI are shown in Table 3. There were no differences in any parameter, based on severity of AKI, at any time point.

**Table 3:** Systemic and regional haemodynamic variables

Timepoint	D0	D1	D2	D4
*Cardiac Index (L/min/m3)*				
HC	2.73 (2.25–2.87)			
Severe	3.24 (2.35–3.84)	3.16 (2.72–3.59)	3.25 (2.44–3.94)	3.56 (2.53–3.94)
Non-severe	2.89 (2.61–3.3)	3.07 (2.84–3.74)	2.95 (2.84–3.2)	3.35 (3.13–3.65)
*TAPSE (cm)*				
HC	2.62 ± 0.24			
Severe	1.91 ± 0.60*	2.15 ± 0.55	2.28 ± 0.45	2.16 ± 0.47
Non-severe	2.0 ± 0.33*	2.36 ± 0.50	2.12 ± 0.40	2.18 ± 0.41
*Pulmonary artery systolic pressure (mmHg)*				
HC	26 (23–30)			
Severe	36 (29–49)*	39 (32–46)	41 (26–50)	40 (31–51)
Non-severe	37 (28–41)*	32 (29–39)	33 (30–40)	33 (31–34)
*IVC CI*				
HC	0.47 (0.37–0.54)			
Severe	0.15 (0.11–0.22)*	0.18 (0.10–0.29)	0.22 (0.15–0.29)	0.23 (0.15–0.39)
Non-severe	0.22 (0.19–0.34)*	0.27 (0.15–0.34)	0.35 (0.21–0.49)	0.30 (0.24–0.37)
*CVP (mmHg)*				
Severe	13.1 ± 5.6	11.4 ± 4.88	12.7 ± 5.4	12.7 ± 4.7
Non-severe	10.5 ± 4.3	11.4 ± 4.9	8.9 ± 4.6	8.6 ± 2.9
*Renal Blood Flow (L/min)*				
HC	1.16 (0.90–1.36)			
Severe	1.33 (0.89–2.04)	1.37 (0.99–1.99)	1.3 (0.88–1.42)	1.18 (0.93–1.57)
Non-severe	1.36 (1.26–1.97)	1.53 (1.1–2.03)	0.86 (0.78–1.58)	1.36 (1.06–1.9)
*Renal RI*				
HC	0.6 (0.56–0.65)			
Severe	0.8 (0.76–0.86)*	0.79 (0.75–0.85)	0.81 (0.73–0.87)	0.80 (0.72–0.83)
Non-severe	0.83 (0.74–0.86)*	0.78 (0.74–0.83)	0.80 (0.76–0.84)	0.8 (0.76–0.82)

*p < 0.05 vs. HC, #*p* < 0.05 between patient groups

### Sublingual microcirculation and markers of tissue perfusion

Sublingual microcirculatory variables and surrogate markers of tissue perfusion (lactate, central venous oxygen saturation, CO_2_ gap) are shown in Table 4. Patients with severe AKI had evidence of impaired microcirculatory flow (MFI 2.55 a.u. (2.17–2.82)) at the initial time point but this resolved in the first 24 h. There were no differences in any sublingual microcirculatory variable based on the degree of AKI. Initial lactate concentration was higher in the group with severe AKI but this difference did not persist past the initial time point. There were no notable abnormalities in either central venous oxygen saturation or CO2 gap in either group at any time point.

**Table 4:** Sublingual microcirculatory variables and surrogate markers of tissue perfusion

Timepoint	D0	D1	D2	D4
*Total Vessel Density (mm/mm*^*2*^*)*				
Severe	17.0 ± 3.16	17.3 ± 4.12	18.0 ± 2.70	17.9 ± 2.96
Non-severe	18.2 ± 2.78	17.8 ± 3.20	17.5 ± 3.64	15.7 ± 2.51
*Perfused Vessel Density (mm/mm*^*2*^*)*				
Severe	14.8 (11.8–16.7)	16.2 (12.9–18.6)	16.8 (14.6–18.1)	16.8 (14.3–19.0)
Non-severe	14.4 (12.9–19.1)	14.4 (12.9–19.1)	15.8 (13.1–19.1)	14.3 (12.0–16.8)
*Microcirculatory Flow Index (a.u.)*				
Severe	2.55 (2.17–2.82)	2.76 (2.61–2.86)	2.79 (2.59–2.9)	2.84 (2.77–2.91)
Non-severe	2.74 (2.54–2.81)	2.74 (2.63–2.81)	2.83 (2.71–2.91)	2.77 (2.66–2.98)
*Microcirculatory Heterogeneity Index (a.u.)*				
Severe	0.3 (0–0.78)	0 (0–0.36)	0 (0–0.17)	0 (0–0.09)
Non-severe	0.14 (0–0.60)	0 (0–0.13)	0 (0–0.02)	0 (0–0.27)
*Lactate (mmol/L)*				
Severe	3.9 (2.72–7.35)	2.3 (1.7–3.8)	1.9 (1.4–2.8)	1.6 (1.1–2.1)
Non-severe	2.7 (1.9–3.5)*	1.6 (1.3–1.7)	1.3 (1.1–1.5)	1.1 (1.0–1.4)
*ScvO2 (%)*				
Severe	70.6 (62.4–76.7)	71.5 (65.6–77.5)	70.1 (66–77.3)	67.3 (62.5–73.2)
Non-severe	70.4 (65.4–73.1)	70.3 (64.4–75.5)	70.1 (65.5–72.8)	60 (58.9–69.4)
*ScvCO2-SaCO2 (kPa)*				
Severe	0.89 (0.54–1.16)	0.67 (0.54–0.92)	0.6 (0.5–0.84)	0.77 (0.58–1.04)
Non-severe	0.88 (0.75–1.11)	0.85 (0.74–0.91)	0.76 (0.66–0.83)	0.67 (0.57–0.89)

AKI + (KDIGO 3), AKI–(KDIGO 0–2), *p < 0.05 between patient groups

Linear regression analysis for selected CEUS variables (mean transit time and perfusion index) and markers of macro- and micro-circulation and surrogates of tissue perfusion are shown in Table 5. There was a weak relationship between lactate and CEUS markers of perfusion but no other associations were observed.

**Table 5:** Correlation (Spearman) between CEUS and other variables for all patients at all timepoints

	mTT (sec)		PI (a.u.)	
	Correlation coefficient (R)	*p* value	Correlation coefficient (R)	*P* value
Cardiac Index	−0.18	< *0.05*	0.2	< *0.05*
MFI	−0.16	< *0.05*	0.18	< *0.05*
PVD	0.02	*0.82*	−0.06	*0.48*
Lactate	0.31	< *0.001*	−0.33	< *0.001*
ScvO2	−0.12	*0.11*	0.04	*0.62*
CO2 gap	0.15	< *0.05*	−0.04	*0.58*

Italics are used to identify the significance values

Correlation coefficients were calculated for both MFI and cardiac index and PVD and cardiac index. Neither demonstrated a significant correlation, correlation coefficient (R) 0.07 (*p* = 0.06) and 0.03 (*p* = 0.334), respectively.

## Discussion

For the first time, in a clinical setting, we report an assessment of blood flow and perfusion in the kidney and in a separate microcirculatory bed, alongside measurements of regional and systemic macrovascular blood flow. The principal finding of the current study is that in patients with septic shock the severity of acute kidney injury was related to the degree of renal cortical hypoperfusion and that this hypoperfusion did not appear to be associated with alterations in regional or systemic blood flow.

Our findings build on previous experimental and clinical studies. Lima and colleagues, using a porcine model of induced sepsis, demonstrated that both renal and sublingual microcirculatory impairment persisted after normalisation of cardiac output [6]. However, the CEUS model used by these investigators relied on bolus administration of contrast and also predominantly reported intensity-based variables to describe cortical perfusion, limiting comparisons with the current study. Harrois and colleagues described reduced renal cortical perfusion in septic shock patients when compared to other critically ill patients but were unable to demonstrate significant differences based on the presence or absence of AKI in their small cohort [7].

Our findings suggest that although microcirculatory impairment was initially apparent in both the renal and sublingual microcirculations, those in the kidney were both more severe and persistent. The finding that other clinically utilised markers of systemic hypoperfusion, such as lactate, central venous oxygen saturations and venous—arterial CO_2_ differences normalised rapidly and did not differentiate between AKI severity suggest that the principal mechanism driving cortical hypoperfusion may be, at least partially, intrinsic to the renal circulation. These findings, alongside those showing no correlation between renal cortical perfusion and either cardiac output or renal blood flow, are clinically important suggesting that any attempt at preventing or ameliorating the effects of AKI in septic patients' needs to focus on blood flow within the renal cortex itself, as therapies directed at other endpoints may prove ineffective. We also noted marked heterogeneity of cortical blood flow, even in patients with similar degrees of AKI, suggesting that therapeutic interventions would be best targeted on an individual patient basis rather than in a “one size fits all” approach.

The apparent lack of correlation between alterations in renal cortical blood flow and changes in the sublingual microcirculation circulation further suggest that the mechanism of cortical hypoperfusion observed in septic AKI may be at least partially caused by factors intrinsic to the kidney rather than a feature of a more generalised endotheliopathy. Histopathological changes reported in septic AKI are also less than expected, particularly given the severe clinical phenotype often observed, which further suggests a predominately functional cause [18, 19]. The microvascular anatomy of the kidney is unique, and one possible explanation for the findings of the current study are the recruitment of shunts within the renal vasculature. Such juxtaglomerular bypass mechanisms would reduce glomerular filtration rate but maintain renal blood flow. Precisely why the kidneys of some patients react to a septic insult in this way is unclear. In 1990, Schurek described pre-glomerular shunts within the rat cortex between interlobular vessels [20]. Shunting may allow flow within the renal artery to be maintained while reducing blood volume within the cortex and reducing GFR, particularly during injury and repair and may represent an adaptive process, potentially reducing exposure to reactive oxygen species [21]. In pathological conditions such as sepsis, increased shunting may lead to medullary hypoxia, while insufficient shunting may result in oxidative stress [22]. Whatever the evolutionary explanation such a mechanism may be maladaptive in sepsis-induced AKI and a potential target for therapeutic intervention. One such approach could investigate the cortical perfusion changes with selective vasopressors and whether doses or agents may be used to optimise perfusion and assessed serially using CEUS studies. Certain pharmacological agents, such as vasopressin and angiotensin 2, are purported to selectively alter afferent and efferent arteriolar tone [23–26] and the use of them in patients at risk of septic AKI, evaluated by CEUS imaging would be an interesting future study.

In addition to the afferent and efferent arteriolar tone, renal perfusion pressure could contribute to alterations in cortical perfusion. In the present study, patients with severe AKI were on higher doses of noradrenaline than those in whom it did not develop. Whether noradrenaline reduces cortical perfusion is unclear. Schneider et al. studied the renal effects of noradrenaline using CEUS and demonstrated marked heterogeneity, some patients demonstrated improved perfusion, while others worsened [14]. Noradrenaline is also known to be a potent venoconstrictor, and it is possible noradrenaline contributed to reduced cortical perfusion by increasing the pressure of venous return [27]. In the present study, central venous pressure tended to be higher in the severe AKI group throughout the study period. Elevated CVP is a risk factor for AKI along with a positive fluid balance [28] and the role of venous congestion in the pathogenesis of septic AKI is the subject of continued investigation. After the commencement of this study, Beaubien-Souligny described ultrasonographic parameters which identify venous congestion and predict AKI following cardiac surgery. The combined assessment of venous congestion and cortical perfusion could form the premise for further study and has the potential to add to the mechanistic understanding of this important disease process [29, 30].

CEUS for the assessment of renal perfusion in critically ill patients is a new and emerging technique which has the potential to allow investigators a real-time window on renal perfusion, allowing rapid assessment of the efficacy of therapeutic interventions in individual patients. Perfusion CEUS has been adapted from a technique used to delineate vascular structures in solid organs and as such the variables reported in the present study are relatively novel. As perfusion CEUS becomes increasingly used as a research tool investigators should seek consensus on the standardisation of image acquisition and analysis.

We suggest that infusion with destruction-replenishment kinetics should become the standard technique in the assessment of tissue perfusion, as opposed to bolus administration of contrast which generates indicator dilution curves with significant confounding influences from global and regional blood flow. Of the CEUS variables recorded in the current study those based on intensity such as relative blood volume are limited by multiple patient and technical factors and have previously performed poorly in reliability studies, with intra and inter-individual variability [31]. In the present study, RBV was the only variable to demonstrate a parametric distribution, a different distribution to the remaining variables. It was the only variable where healthy control data centred closely to the median patient value, and if controls are assumed to have better perfusion than patients, which the other variables suggest, then RBV may be non-discriminatory for identifying hypoperfusion within a tissue bed. It is possible that RBV represents a measurement comparable to that of total vessel density measured with handheld video microscopy. TVD is a measure of red cell density within a tissue bed which may be analogous to the bubble concentration quantified by RBV. TVD is influenced by the anatomical density of vessels within a tissue bed [16] and only reduces after sufficient capillary segments have no flow, a behaviour similar to RBV, which only became abnormal in those with more severe reductions in renal perfusion.

Perfusion index and wash-in rate demonstrated extreme collinearity in the present study, and we suggest that one variable would therefore suffice in future studies. Mean transit time is less dependent on factors such as organ depth and gain settings [31]. As expected, mTT values were lowest in healthy controls, elevated in patients who did not develop severe AKI and most elevated in those with AKI.

We were unable to demonstrate associations between macrovascular variables such as cardiac index and microcirculatory flow in either the renal or sublingual microcirculation. To our knowledge, this is the first clinical study to demonstrate haemodynamic incoherence of two separate regions and strengthens the argument supporting independence of large vessel flow and tissue oxygen delivery in shocked states [32].

Our study has several limitations. Although the sample size was larger than any previous clinical study, it was conducted at a single site on a relatively small number of patients. We had to change our a priori outcome from AKI or no AKI after the vast majority of patients developed at least KDIGO stage 1 AKI. Furthermore, our study cohort was heavily skewed towards those with KDIGO 3 AKI, principally due to the early commencement of renal replacement therapy following admission to critical care. We included healthy controls but did not include data from non-septic critically ill patients. The healthy control data were essential to define normal values with this exploratory method, and we accept the results are therefore specific to septic AKI. Measurement of medullary blood flow is inherently difficult due to the complex nature of large vessels in the vicinity of regions with critical perfusion. As such we could not confidently report medullary perfusion alterations and the study of this important tissue bed requires further work. The variables we have used to measure cortical perfusion are relatively exploratory and correlation to a gold standard, such as functional MRI would help validate this technique. Finally, the use of ultrasound, as opposed to direct invasive measurement, to assess renal blood flow has been criticised; we attempted to overcome this limitation by utilising the presence of contrast medium to more accurately measure the diameter of the renal artery. In addition, we calibrated our method using healthy volunteers; the mean measured RBF in the volunteer cohort and the assumed normal value for RBF of 1.2 l/min were used to generate a constant. The constant was then multiplied by the measured RBF in the patient cohorts; however, we accept that this is an unproven technique.

## Conclusion

Renal cortical hypoperfusion is a persistent feature in critically ill septic patients who develop AKI and does not appear to be caused by reductions in macrovascular renal blood flow or cardiac output. Cortical hypoperfusion appears not be associated with changes in the sublingual microcirculation, raising the possibility of a specific renal pathogenesis that may be amenable to therapeutic intervention.

## Data Availability

The dataset used for analysis is available from the corresponding author on reasonable request.

## Abbreviations

AKI: Acute kidney injury
BSE: British society of echocardiography
CEUS: Contrast-enhanced ultrasound
CI: Collapsibility index
CKD: Chronic kidney disease
HC: Healthy controls
IDF: Incident dark field
IVC: Inferior vena cava
KDIGO: Kidney disease improving global outcomes
MFI: Microcirculatory flow index
MHI: Microcirculatory heterogeneity index
MI: Mechanical index
mTT: Mean transit time
PaCO2: Arterial partial pressure carbon dioxide
PcvCO2: Central venous partial pressure carbon dioxide
PVD: Perfused vessel density
PI: Perfusion index
RI: Resistive index
RBF: Renal blood flow
SOFA: Sequential organ failure assessment
ScvO2: Central venous oxygen saturation
TAPSE: Tricuspid annular plane systolic excursion
TIC: Time intensity curve
TVD: Total vessel density
WiR: Wash-in rate
